# Dynamic cellular phenotyping defines specific mobilization mechanisms of human hematopoietic stem and progenitor cells induced by SDF1α versus synthetic agents

**DOI:** 10.1038/s41598-018-19557-x

**Published:** 2018-01-30

**Authors:** Cornelia Monzel, Alexandra S. Becker, Rainer Saffrich, Patrick Wuchter, Volker Eckstein, Anthony D. Ho, Motomu Tanaka

**Affiliations:** 1 Physical Chemistry of Biosystems, Institute of Physical Chemistry, Heidelberg University, 69120 Heidelberg, Germany; 2 Department of Medicine V, Heidelberg University, 69120 Heidelberg, Germany; 3 Institute for Integrated Cell-Material Sciences, Kyoto University, 606-8501 Kyoto, Japan; 4 Present Address: Laboratoire Physico-Chimie, Institut Curie, CNRS UMR168, 75005 Paris, France; 5 Present Address: Institute of Transfusion Medicine and Immunology, Medical Faculty Mannheim, Heidelberg University, German Red Cross Blood Service Baden-Württemberg – Hessen, 68167 Mannheim, Germany

## Abstract

Efficient mobilization of hematopoietic stem and progenitor cells (HSPC) is one of the most crucial issues for harvesting an adequate amount of peripheral HSPC for successful clinical transplantation. Applying well-defined surrogate models for the bone marrow niche, live cell imaging techniques, and novel tools in statistical physics, we have quantified the functionality of two mobilization agents that have been applied in the clinic, NOX-A12 and AMD3100 (plerixafor), as compared to a naturally occurring chemokine in the bone marrow, SDF1α. We found that NOX-A12, an L-enantiomeric RNA oligonucleotide to SDF1, significantly reduced the adhesion of HSPC to the niche surface mediated via the CXCR4-SDF1α axis, and stretched the migration trajectories of the HSPC. We found that the stretching of trajectories by NOX-A12 was more prominent than that by SDF1α. In contrast, plerixafor exhibited no detectable interference with adhesion and migration. We also found that the deformation of HSPC induced by SDF1α or plerixafor was also drastically suppressed in the presence of NOX-A12. This novel technology of quantitative assessment of “dynamic phenotypes” by physical tools has therefore enabled us to define different mechanisms of function for various extrinsic factors compared to naturally occurring chemokines.

## Introduction

Functions of somatic stem cells are strictly governed by an appropriate balance between self-renewal and differentiation. This balance is in turn regulated by interactions between stem cells and their microenvironment-the so-called “niche”. In the case of hematopoietic stem and progenitor cells, the dormancy of the most primitive HSPC is maintained by the bone marrow niche by means of several key molecular interactions between receptor-ligand pairs^1–3^. For example, it has been suggested that homophilic, N-cadherin-mediated adhesion between HSPC and mesenchymal stem cells (MSC) supports long-term maintenance of the primitive HSPC pool^4–6^. Another key molecular axis is the interaction between stromal cell-derived factor 1α (SDF1α or CXCL12) and its receptor CXCR4, expressed on the cell surface of HSPC. This axis plays a significant role in homing and migration of HSPC^7–15^.

In recent years, peripheral HSPC have largely replaced bone marrow-derived cells for autologous transplants, and they have become the major source of stem cells also for allogeneic transplantations^16–21^. Efficient mobilization of HSPC is a prerequisite for the successful stem cell collection and consecutive transplantation. G-CSF, the standard and most widely used agent for this purpose over the past 25 years, mobilizes stem cells from the marrow niche by secretion of neutrophil-associated extracellular proteases which subsequently releases HSPC from their niche^22,23^.

About 10–15% of patients intended for autologous transplantation have difficulties in mobilizing an adequate amount of HSPC for transplantation^24^. In this case, new and highly effective mobilizing reagents are needed. For example, plerixafor (AMD3100)^25,26^ has been proven highly effective for the mobilization of CD34^+^ cells for autologous transplantations, especially in poor mobilizing patients^27–35^. Initially regarded as a CXCR4-antagonist, the mechanism of action of plerixafor might be more complex and, according to recent evidence, even as a partial agonist^10,11,13^. NOX-A12 (NOXXON Pharma), an L-enantiomeric RNA oligonucleotide, also targets the CXCR4-SDF1α axis by binding and neutralizing SDF1α. This compound showed a half-maximal inhibitory concentration value of 300 pM (4.3 ng/mL) in a migration assay using Jurkat cells^36^.

In addition to mobilizing HSPC, the interference with the CXCR4-SDF1α axis has also been proposed as a possible strategy to “mobilize” malignant stem cells from their protective niche, thus rendering tumor stem cells more vulnerable to chemo- or irradiation therapy. Several studies indicated that intimate contact between CXCR4 expressed on tumor cells and SDF1α in the niche might represent a key mechanism for metastatic spread and tumor resistance^37,38^. Hoellenriegel *et al*. reported that NOX-A12 induced the release of CXCL12 from murine stromal cell lines, and inhibited chemotaxis of chronic lymphatic leukemia cells and lymphoid cell lines^39^.

How these aforementioned molecules interfere with the CXCR4-SDF1α axis or how their mechanisms of action are different from those of SDF1α has yet to be defined. For example, both plerixafor and SDF1α generate phosphorylation of MEK1/2 and ERK1/2, resulting in cell proliferation. However, plerixafor leads to a sustained release of cAMP, while SDF1α causes a surge of cAMP. NOX-A12 was designed as a “Spiegelmer” to neutralize SDF1α in a similar manner to antibodies, but the molecular mechanism of NOX-A12 on HSPC mobilization is largely unknown.

**Figure 1:**
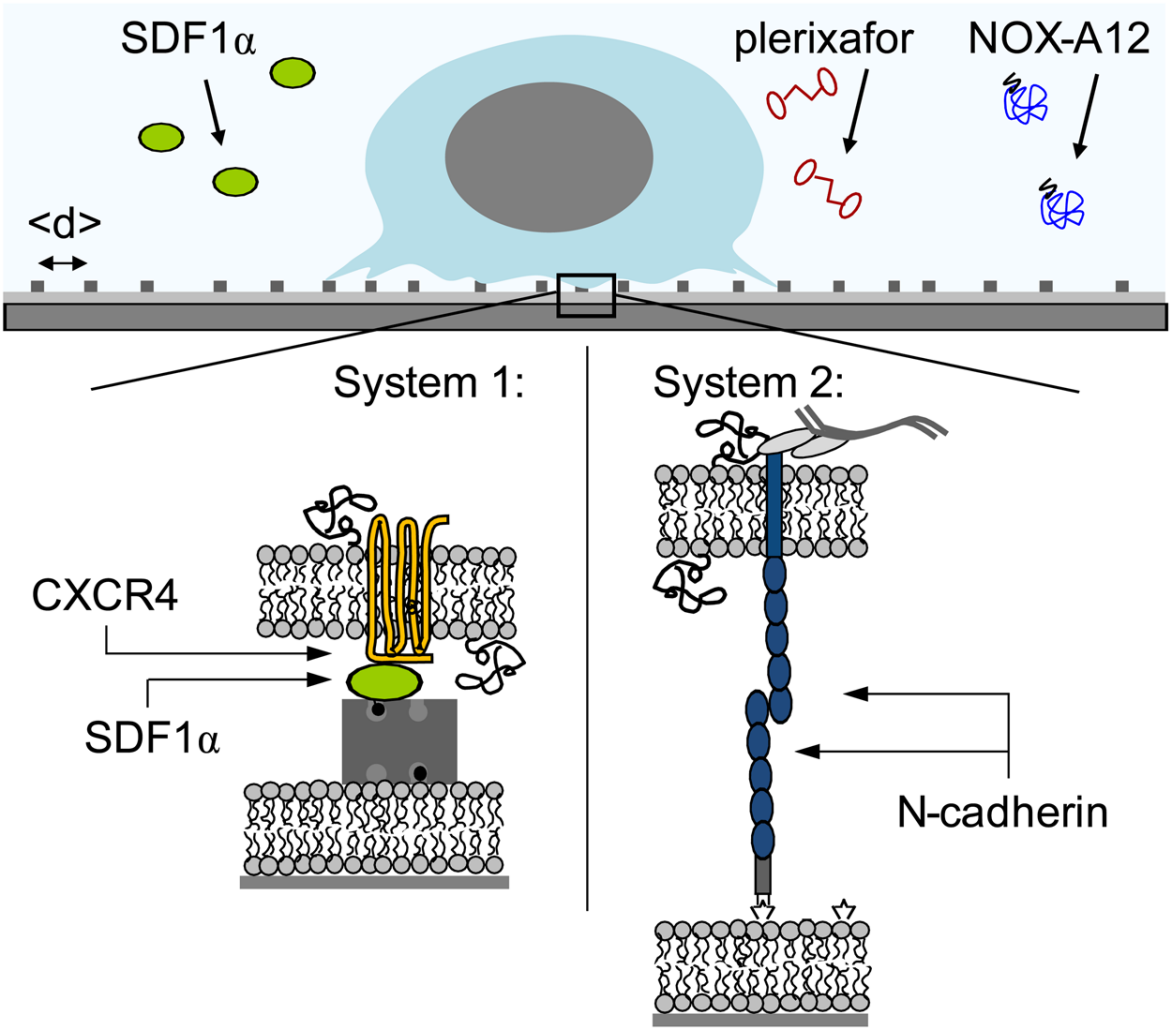
Schematic illustration of HSPC adhering to *in vitro* surrogate surfaces based on planar lipid membranes (supported membranes) displaying SDF1α or N-cadherin axis. Influence of NOX-A12 or plerixafor in the medium on the adhesion, active deformation and migration of HSPC was compared to SDF1α.

In this study, we have quantitatively assessed the impact of NOX-A12 and plerixafor versus SDF1α on the adhesion, migration, as well as on dynamic deformation of HSPC by using defined surrogate niche models, based on planar lipid membranes on solid substrates (called supported membranes, Fig. 1)^40^. By controlling the self-assembly of lipid anchors with biotin or nitro-triacetic acid (NTA) head groups, it is possible to precisely control the intermolecular distance between the ligand molecules on the membrane surfaces in nm accuracy^41,42^. We have recently fabricated the surrogate niche surfaces based on supported membranes displaying SDF1α or N-cadherin, and quantified the strength of healthy HSPC versus CD34^+^ leukemia blasts from acute myeloid leukemia patients^43^. Moreover, by means of physical and thus quantitative assays, we have demonstrated that the adhesion and migration of HSPC on the surrogate surface was altered by the presence of physiological SDF1α molecules in solutions (5 ng/mL). In continuation of our “physical phenotyping” strategy, we have defined the dynamic behavior of HSPC upon exposure to NOX-A12 or plerixafor, two compounds that are of high clinical relevance, and compared it to naturally occurring SDF1α.

## Results and Discussion

### Impact on HSPC-niche interaction mediated via SDF1α-CXCR4 axis

**Figure 2:**
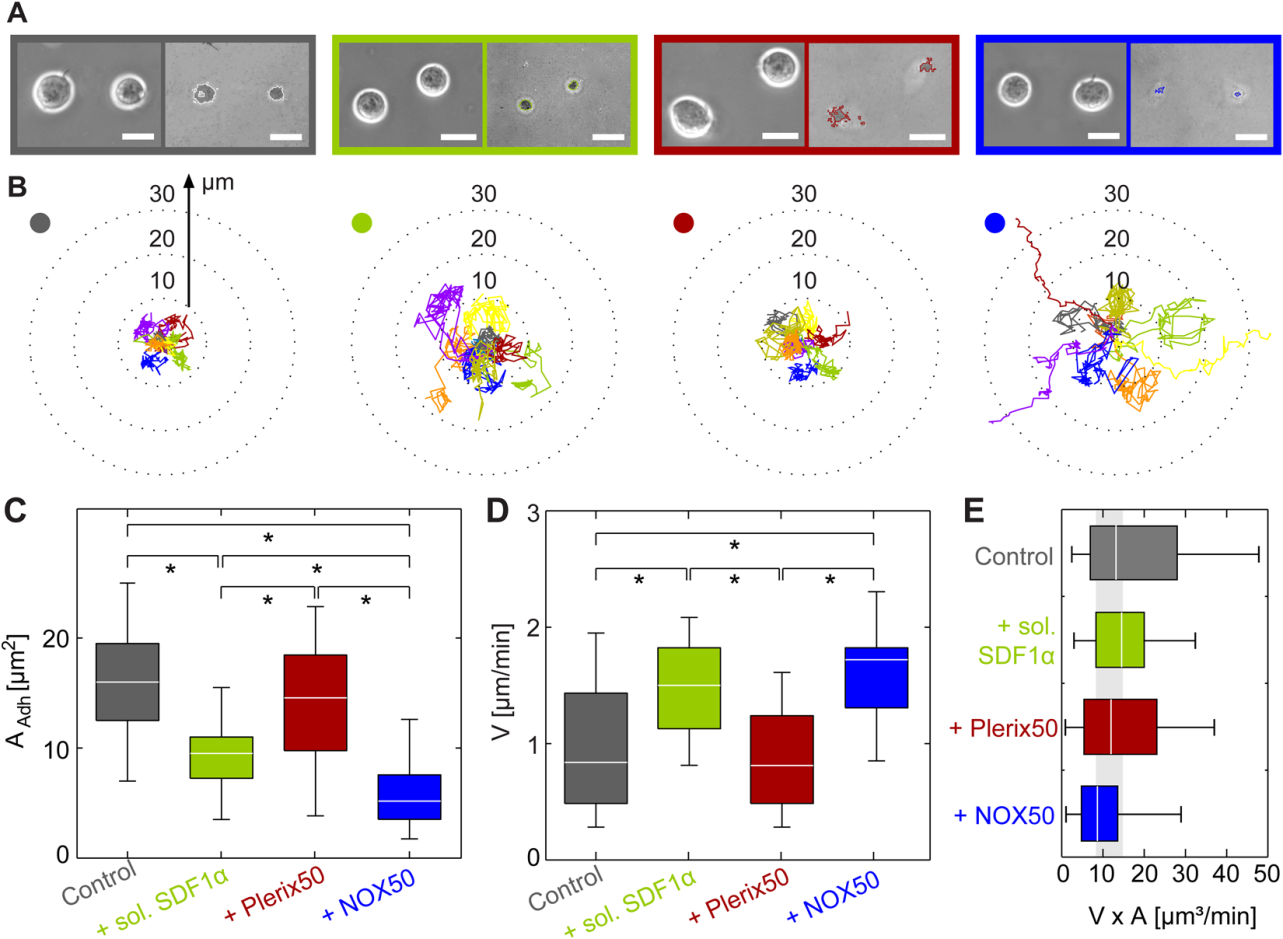
(**A**) Phase contrast (left) and RICM images (right) of HSPC adhering to SDF1α in absence (grey) and presence of sol. SDF1α (green), plerixafor (red, Plerix50) and NOX-A12 (blue, NOX50). Scale bar 5 µm. (**B**) Migration trajectories of HSPC recorded over 1 h, (**C**) cell adhesion area *A*, (**D**) migration velocity, *V*, and (**E**) *V* × *A* on SDF1α substrates. The colored dots in (**B**) correspond to the different sample treatments. Significance levels p < 0.001 evaluated by Mann-Whitney U test as indicated by (*).

Figure 2A shows the adhesion behavior of HSPC to the surrogate niche model displaying SDF1α as the ligand. Four sets of a phase contrast image (left) and a RICM image (right) of HSPC adhering on the surrogate surfaces with SDF1α at an intermolecular distance of <*d*> = 11 nm. The phase contrast images represent the global morphology of HSPC, while RICM images highlight the shape of adhesion zones^43^. The experiments were performed in the absence of soluble factors (control, grey) and in the presence of following factors: 5 ng/mL SDF1α (green), 50 ng/mL plerixafor (red), and 50 ng/mL NOX-A12 (blue). The concentration of SDF1α adopted for the experiments corresponds to the physiological level in human bone marrow. The concentration of plerixafor, 50 ng/mL (corresponding to ≈0.1 µM) was chosen by consulting previous *in vitro* studies using 500 ng/mL^11^. In the case of NOX-A12, this concentration level (≈3.5 nM) was between the IC50 level found in *in vitro* chemotaxis study on chronic lymphatic leukemia cells and lymphoid cell lines (≈0.3 nM)^39^ and the plasma level at which effective mobilization of leukocytes in human was observed (~1 µM)^44^. HSPC were incubated for 2 h with the respective soluble factors and allowed to adhere onto the surrogate surfaces for 1 h. RICM images (right) suggest that the adhesion area per cell *A* significantly decreased in the presence of SDF1α (green) and NOX-A12 (blue) compared to the control experiments (grey), but plerixafor (red) induced almost no detectable change. Figure 2B represents the migration trajectories of HSPC in the presence and absence of soluble factors. Each trace corresponds to a trajectory monitored for 1 h. The trajectories in the presence of plerixafor (red) were as compact as the control ones (grey), and the trajectories hardly exceeded radial distance of 10 µm from the initial position. The presence of SDF1α (green) led to a clear extension of the trajectories, which were even more pronounced in NOX-A12 (blue). In the presence of NOX-A12, some trajectories were extended >30 µm from the initial position.

Figure 2C shows the statistical comparison of the adhesion area *A* calculated from >30× cells for each conditions. The median value of adhesion area for the control sample (grey) *A*_control(SDF)_ = 16 µm^2^. In the presence of 5 ng/mL SDF1α, *A* exhibited a clear decrease to *A*_SDF(SDF)_ = 10 µm^2^. Interestingly, plerixafor (red) did not cause any remarkable change, *A*_plerixafor(SDF)_ = 15 µm^2^. This finding clearly indicates that plerixafor does not act as an antagonist that directly interferes with the CXCR4-SDF1α axis. The incubation with NOX-A12 (blue) resulted in a significant decrease in the adhesion constant by a factor of 3, *A*_NOX(SDF)_ = 5.2 µm^2^. It is notable that the decrease in *A* caused by NOX-A12 is more prominent compared to that caused by SDF1α in solution. This finding supports the notion that SDF1α in solution shifts the equilibrium between “bound” and “unbound” SDF1α and thus reduces the fraction of “bound” CXCR4-SDF1α complexes, but does not cause cell detachment. NOX-A12 is an RNA oligomer in L-configuration, which binds and neutralizes SDF1α. Therefore, NOX-A12 does not only shift the equilibrium but also neutralizes SDF1α on the surrogate surfaces, resulting in the weakest HSPC adhesion. Last but not least, we found no sign of cell detachment or cell death (apoptosis) throughout the time-lapse imaging over 6 h.

The elongation of the migration trajectories observed in the presence of SDF1α and NOX-A12 (Fig. 2B) suggest remarkable modulations of the persistency and velocity of HSPC migration. Figure 2D represents the migration velocity *V* measured under the four conditions. The migration velocity of HSPC in the presence of SDF1α (*V*_SDF(SDF)_ = 1.5 µm/min) was almost two times higher than that of the control (*V*_control(SDF)_ = 0.85 µm/min), which seemed to coincide with the native function of chemokine SDF1α. The migration velocity in the presence of plerixafor remained comparable to the range of control levels, *V*_plerixafor(SDF)_ = 0.82 µm/min. The fact that plerixafor did not induce any increase in the migration velocity seemed contradictory to the previous reports demonstrating that plerixafor alone could mobilize HSPC *in vivo*. However, it should be noted that the HSPC mobilization *in vivo* was assessed by the increase in HSPC in peripheral blood but not by the migratory velocity. In fact, more recent studies provided evidence that plerixafor acts not only as an antagonist of SDF1α-CXCR4 but also as a partial agonist^10,11,13,15^. As presented in Fig. 2C, the adhesion area under exposure to plerixafor was comparable to the controls, suggesting that plerixafor did not directly interfere with the SDF1α-CXCR4 axis. In contrast, NOX-A12 seemed to directly interfere with adhesion and migration of HSPC mediated via SDF1α-CXCR4. The area of adhesion in the presence of NOX-A12 was 3 times less than the controls, and the migration velocity in the presence of NOX-A12 *V*_NOX(SDF)_ = 1.8 µm/min was even faster than that in SDF1α solution.

When cells migrate at a constant velocity *v*, the frictional force exerted on HSPC follows *F*_*fsrict*_ = *σv*, where *σ* is the frictional coefficient. Since the surrogate surface displays membrane-anchored SDF1α uniformly, the friction exerted on HSPC was linearly proportional to the contact area, *σ* ∝ *A*. As presented in Fig. 2E, *A* and *v* shows a reciprocal relationship, which suggests that the frictional force exerted on HSPC migrating at a constant velocity was comparable under all four conditions.

The results can be summarized as follows. First, the incubation with either SDF1α or NOX-A12 resulted in a reduction in adhesion area and also an increase in the migration velocity compared to the controls. The impact was much more prominent for NOX-A12, implying that SDF1α only shifted the equilibrium between “bound” and “unbound” states but NOX-A12 bound and neutralized SDF1α both in solution and on surrogate surfaces. In contrast, plerixafor induced no major change in adhesion area nor in migration velocity, suggesting that plerixafor did not directly interfere with the SDF1α-CXCR4 axis as an antagonist. This finding is compatible with recent reports in the literature, indicating that plerixafor might act as a partial agonist^11,13,15^.

How can we discriminate differential impacts of SDF1α and the aforementioned agents on the dynamic behavior of HSPC? In general, a round cell can undergo migration by breaking symmetry, characterized by the emergence of higher modes of deformations such as elliptic deformation (m = 2) and front-rare symmetry (m = 3). The shape deformation of a cell is an active process driven by energy consumption, especially associated with remodeling of cytoskeletons and re-organization of cell membranes^45,46^.

**Figure 3:**
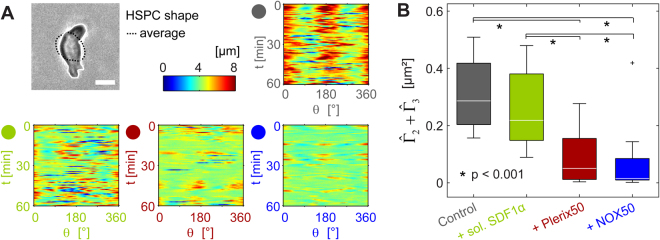
(**A**) Deformation maps of HSPC deformation over time in absence and presence of sol. SDF1α, plerixafor (Plerix50) or NOX-A12 (NOX50) for HSPC on SDF1α functionalized substrates. Scale bar 5 µm. (**B**) Total power of cell deformation from *m* = 2 and 3, ${\hat{{\rm{\Gamma }}}}_{2}+{\hat{{\rm{\Gamma }}}}_{3}$. Significance levels according to Mann-Whitney U test as indicated by (*).

As a first step, the periphery of a cell was determined from each phase contrast image based on a pixel intensity threshold (Fig. 3A). To assess the deformation, the radial distance from the center of mass is plotted in a polar coordinate over 1 h for each condition. The deformation of controls (grey) exhibited strong intensity at *θ* = 0 and ±180 °, suggesting that HSPC underwent the linear elongation. In the presence of SDF1α and drugs in solutions, the radial distance maps became much noisier, showing smaller amplitudes of deformation. To extract quantitative information on active cell deformation hidden behind the noise, we Fourier transformed the shape deformation *c*_m_ for mode *m* = 2 and 3 and calculated the power spectrum:

$$\hat{{{\rm{\Gamma }}}_{{\rm{m}}}}=\langle {c}_{m}(t){c}_{-m}(t)\rangle .$$

**Figure 4:**
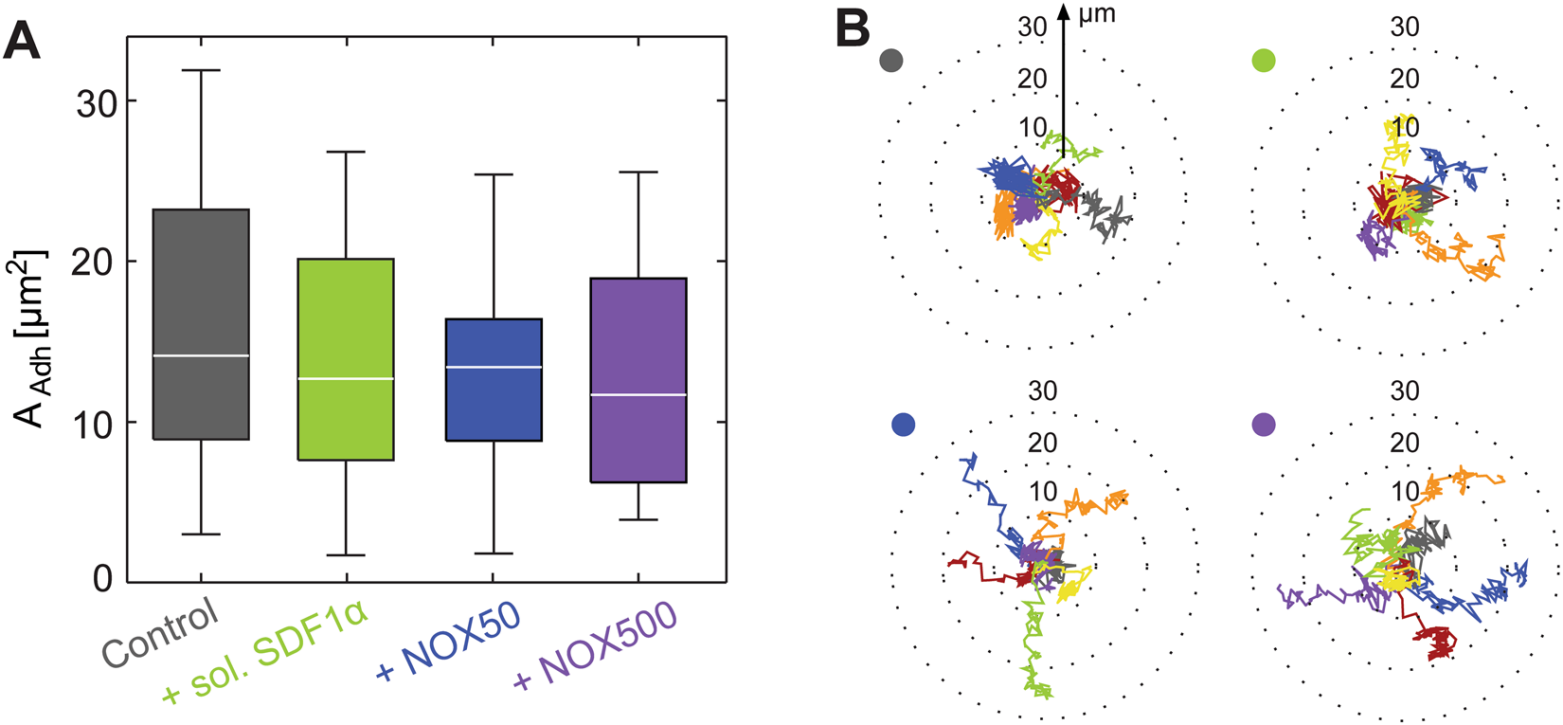
Impact of SDF1α (50 ng/mL) and NOX-A12 (NOX50 (50 ng/mL), and NOX500 (500 ng/mL)) in solutions on (**A**) cell adhesion area ***A***_*Adh*_ and (**B**) migration trajectories of HSPC on surfaces displaying N-cadherin. Mann-Whitney U test revealed no significant changes between data.

Since the center of mass is taken as the origin of inertial frame, the isotropic expansion and contraction (*m* = 0) as well as the translational motion (*m* = 1) were not included. With the aid of the mode analysis of power spectra, we can identify the predominant mode of deformation that HSPC dissipates energy. As HSPC is more compact and less deformable compared to cancer cells^41^, the deformation at higher modes (*m* ≥ 4) is negligibly small^43^. The sum of powers for *m* = 2 and 3 determined from the experimental results, $\hat{{{\rm{\Gamma }}}_{2}}+\hat{{{\rm{\Gamma }}}_{3}}$, is plotted in Fig. 4B. Remarkably, both plerixafor and NOX-A12 resulted in a significant damping of the total power, indicating that both drugs suppressed the active deformation of HSPC. Such an “energy saving” behavior is much less pronounced for SDF1α. As presented in Fig. 3, the influence of SDF1α and NOX-A12 on the adhesion and migration is qualitatively very similar. Both SDF1α and NOX-A12 resulted in a significant decrease in *A*, the extension of trajectories, and an increase in translational velocity *V*, suggesting that they directly interfered with the interactions mediated via SDF1α-CXCR4 axis. On the other hand, plerixafor caused no significant influence on the adhesion and migration of HSPC, indicating that plerixafor did not interfere with SDF1α-CXCR4 interactions. However, the mode analysis in Fourier space implies that plerixafor and NOX-A12 suppressed the energy dissipation driven by active deformation of HSPC, which can hardly be identified in the presence of SDF1α.

Compared to the control samples and in terms of energy dissipation, HSPC responded to external factors in distinctly different patterns: (i) SDF1α interfered with and weakened SDF1α-CXCR4 interactions but did not damp active deformation significantly, (ii) plerixafor did not interfere with SDF1α-CXCR4 interactions but strongly suppressed the active deformation, and (iii) NOX-A12 reduced SDF1α-CXCR4 interactions and strongly suppressed the active deformation. The differential impact of SDF1α versus clinical drugs (plerixafor and NOX-A12) can be attributed to the natural functions of SDF1 as a chemokine to govern HSPC migration in the marrow niche under steady state. Although there was no chemical potential gradient of SDF1α in our experimental system, HSPC underwent active deformation and exhibited extended trajectories. The function of plerixafor was completely different. Plerixafor did not block the binding of SDF1α to CXCR4, but pushed HSPC to the “energy saving mode”. The effect of NOX-A12 on adhesion and migration was completely compatible with the neutralization of SDF1α, although the damping of energy consumption could not be explained by the antagonistic function against SDF1α. Thus, the quantitative data obtained from these physical analyses: adhesion, deformation, and motion of HSPC has enabled us to dissect differential functions of NOX-A12 from plerixafor, as well as from the natural chemokine SDF1α. As a reference, we performed the same series of experiments with peripheral blood HSPC from healthy donors (Supporting Information Fig. S3), and found that the influence of NOX-A12 was comparable between two HSPC types, while plerixafor causes a significant decrease in adhesion area *A*, an increase in migration velocity *V*, and a decrease in deformation power ${\hat{{\rm{\Gamma }}}}_{2}+{\hat{{\rm{\Gamma }}}}_{3}$. The more pronounced “mobilization” of peripheral blood by plerixafor seems plausible, as the peripheral HSPC had been mobilized from the bone marrow with G-CSF^22^. Previous *in vivo* studies have also demonstrated that the combination of plerixafor and G-CSF results in the enhancement of HSPC mobilization^30,47^.

### Impact of NOX-A12 on HSPC-niche interaction mediated via homophilic N-cadherin axis

Subsequently, we examined how NOX-A12 influenced the adhesion, deformation, and migration of HSPC exposed to surrogate surfaces displaying N-cadherin as the ligand (System 2, Fig. 1). As reported previously, N-cadherin-mediated adhesion between HSPC and the marrow niche is crucial for the long-term maintenance of the HSPC pool^4^. As presented in Fig. 4A, the area of HSPC adhesion on the surrogate surface displaying N-cadherin is *A*_control(cad)_ = 14 µm² in the absence of soluble factors. In contrast to the data generated by using SDF1α-functionalized surfaces, the adhesion areas between HSPC and niche surface showed no remarkable change in the presence of 5 ng/mL SDF1α and 50 ng/mL NOX-A12; *A*_SDF(cad)_ = 12.7 µm², *A*_NOX50(cad)_ = 13.4 µm². Actually, the change caused by an increase in NOX-A12 concentration to 500 ng/mL was still minor, *A*_NOX500(cad)_ = 11.7 µm², and hence indicated that further increase in the dose level did not have any significant effect^39^. Our experimental data have provided direct evidence that both SDF1α and NOX-A12 did not interfere with the adhesion mediated by homophilic N-cadherin axis. Although the influence on adhesion area caused by the external factors seemed minor, the migration trajectories exhibited a distinct difference (Fig. 4B). The trajectories in the presence of SDF1α were comparable to those of controls,where the start-to-end distances remained ≤20 µm. On the other hand, the migration trajectories of HSPC in the presence of 50 ng/mL and 500 ng/mL NOX-A12 were more elongated, where 3 out of 7 trajectories in Fig. 4B exhibited the start-to-end distance of >20 µm.

**Figure 5:**
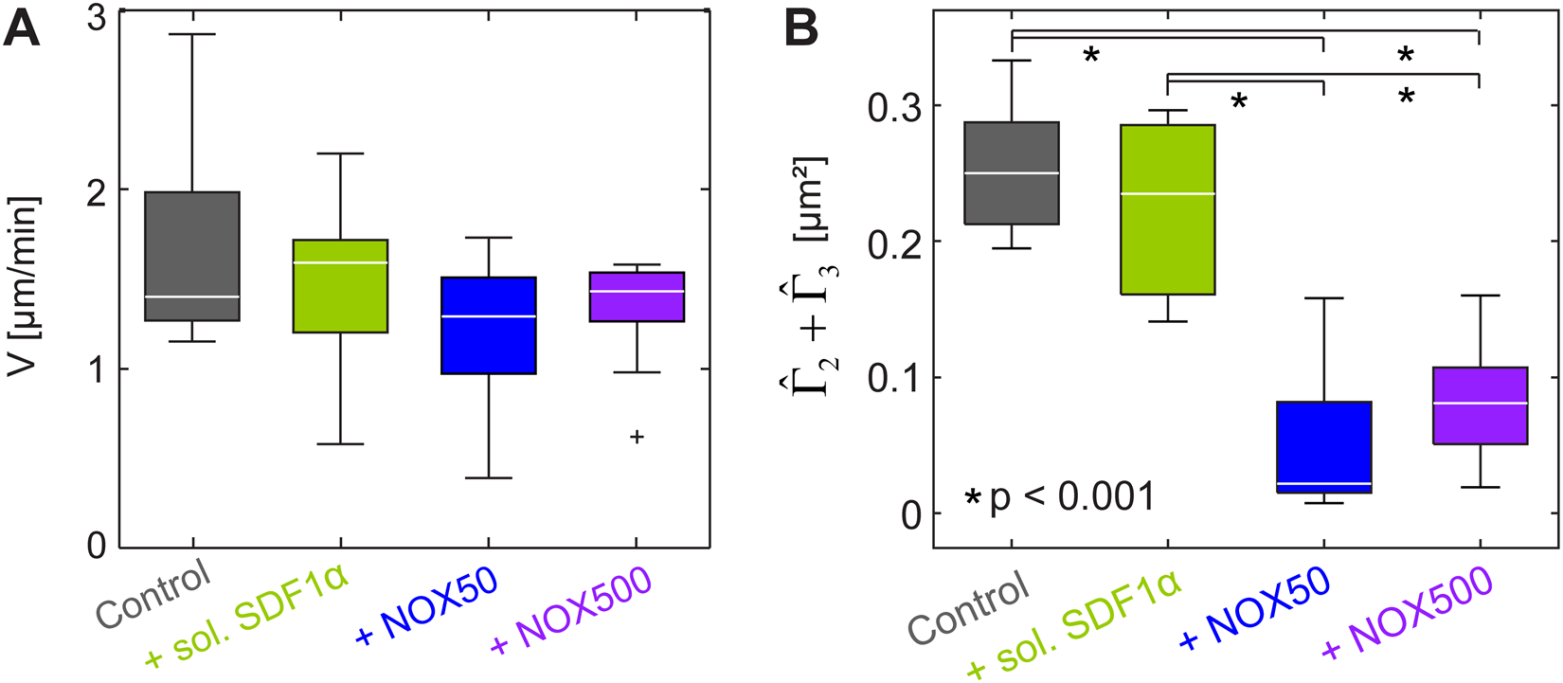
(**A**) Migration velocity *V* and (**B**) total power calculated from m = 2 and 3, ${\hat{{\rm{\Gamma }}}}_{2}+{\hat{{\rm{\Gamma }}}}_{3}$, of HSPC on surrogate surfaces displaying N-cadherin in the presence of soluble SDF1α (50 ng/mL) and NOX-A12 (NOX50 (50 ng/mL), and NOX500 (500 ng/mL)). Significance levels according to Mann-Whitney U test indicated by (*).

Interestingly, such elongation of migration trajectory was not accompanied by an increase in migration velocity (Fig. 5A). The median values for the migration velocity all remained within *V* = 1.3–1.6 µm/s under all conditions. Note that the migration velocity of controls on N-cadherin surface *V*_control(cad)_ = 1.4 µm/s was larger than the corresponding value on SDF1α surface *V*_control(SDF)_ = 0.84 µm/s, which could be attributed to the lower binding affinity of homophilic N-cadherin (*k*_D(N-cadherin)_ ~22 µM^48^) compared to CXCR4-SDF1α axis (*k*_D(SDF1α-CXCR4)_ ~5 nM^49^). Moreover, the fact that both adhesion area *A* and migration velocity *V* were very similar in the absence or presence of soluble factors suggested that the frictional coupling between HSPC and N-cadherin functionalized surfaces was not influenced by the soluble factors (Figure S2). This finding was contradictory to our findings on the surrogate surfaces displaying SDF1α, showing that SDF1α and NOX-A12 directly interfered with the frictional coupling between HSPC and SDF1α coated surfaces.

To define the mechanisms behind the elongation of trajectories induced by NOX-A12, we examined the impact of NOX-A12 on active HSPC deformation by the power spectrum analysis. This analysis indicated that the total power of cell deformation from *m* = 2 and 3, ${\hat{{\rm{\Gamma }}}}_{2}+{\hat{{\rm{\Gamma }}}}_{3}$, was strongly damped in the presence of NOX-A12 (Fig. 5B). This clearly differentiates NOX-A12 from SDF1α, which caused no remarkable change in the deformation power. Thus, the quantitative assays we employed in this study have enabled us to discriminate different functional mechanisms of NOX-A12 from SDF1α on the interactions between HSPC and the marrow niche as mediated by the homophilic N-cadherin axis.

To gain further insight into these experimental findings, a theoretical model describing the deformation and migration of cells would be extremely helpful. Recently, Ohta *et al*. proposed a simple model derived from the symmetry confideration, and demonstrated the nonlinear coupling between the active deformation and motion significantly influence the migration trajectories^50^. At present, this model cannot be adopted to simulate the migration HSPC, since the stochastic deformation forces in their model does not match the experimental observation. Nevertheless, the combination of the experimental dynamic phenotyping and a mathematical model will help us understand the mode of functions for various natural and synthetic agents.

## Conclusions

Our quantitative analysis demonstrated that NOX-A12 directly interferes with adhesion mediated via SDF1α-CXCR4 axis (Fig. 2C). In fact, the adhesion area of HSPC treated with NOX-A12 is reduced by a factor of 2 compared to HSPC exposed to SDF1α in medium, which can be attributed to the neutralization of SDF1α displayed on the surrogate surfaces. Note that the effect of NOX-A12 on SDF1α-CXCR4 axis was completely different from that induced by plerixafor. We have demonstrated that the presence of plerixafor was not associated with any change in the adhesion area, which is in accordance with the results from other authors. Therefore, we concluded that plerixafor does not function only as a pure antagonist to the SDF1α-CXCR4 axis. In contrast, NOX-A12 has no influence on the adhesion mediated via homophilic N-cadherin axis (Fig. 4A). These results are fully understandable from the molecular design of NOX-A12 as a molecule that selectively binds and neutralizes SDF1α.

The migration trajectories of NOX-A12-treated HSPC on SDF1α-functionalized surfaces were significantly elongated compared to the control sample (Fig. 2B). This was associated with a distinct increase in the migration velocity (Fig. 2D). This observation coincided precisely with the effect caused by SDF1α in medium, which can be partially interpreted in terms of a smaller adhesion area. However, the change in adhesion area and thus the frictional coupling between cells and surfaces is not sufficient to explain the difference between NOX-A12 and SDF1α. On surrogate *in vitro* model surfaces displaying N-cadherin, the trajectories in the presence of NOX-A12 were clearly more elongated than those caused by of SDF1α-treated HSPC (Fig. 4B), although the adhesion area did not show any difference (Fig. 4A). These findings suggested that there are additional factors that have an impact on cell migration, such as active cell deformation. The energy dissipation resulting from the active deformation of HSPC can be assessed by the power spectrum analysis for the second and third modes (${\hat{{\rm{\Gamma }}}}_{2}+{\hat{{\rm{\Gamma }}}}_{3}$). An intriguing finding is that the energy dissipation by HSPC treated with NOX-A12 was drastically suppressed compared to that of SDF1α-treated cells on the surfaces functionalized with SDF1α (Fig. 3B) and N-cadherin (Fig. 5B). Since NOX-A12 has no direct binding capability to N-cadherin, our data suggest that NOX-A12 would suppress the dynamic deformation by triggering different molecular pathways independent of the adhesion axis.

In summary, we have demonstrated that the combination of cell adhesion area measurement and migration trajectory analysis, paired with power spectrum analysis represents a powerful tool to define differential functional characteristics of NOX-A12 versus plerixafor, or versus naturally occurring chemokine SDF1α. Such a “dynamic phenotyping” of cells can potentially be used to dissect the influence of different drugs and extrinsic factors on cells, which cannot be extracted otherwise. A thorough understanding of this interplay represents an essential step for the development of new drugs targeting SDF1α–CXCR4 and similar adhesion axes.

## Materials and Methods

### Lipids, proteins

1-stearoyl-2-oleoyl-*sn*-glycero-3-phosphocholine (SOPC), 1,2-dioleoyl-*sn*-glycero-3-[(N-(5-amino-1-carboxypentyl)iminodiacetic acid)succinyl] (nickel salt) (DOGS-NTA (Ni^2+^)) and 1,2-dioleoyl-*sn*-glycero-phospho-ethanolamine-3-N-(cap biotinyl) (biotin-cap-DOPE) were purchased from Avanti Polar Lipids (Alabaster, USA), and neutravidin from Life Technologies. Recombinant stromal cell-derived factor-1 (SDF1α) with and without biotin tags and human N-cadherin with histidin tag were purchased from Almac Group (Craigavon, UK) and R&D Systems Inc. (Wiesbaden, Germany), respectively. Plerixafor was purchased from Sigma and NOX-A12 was provided by NOXXON Pharma AG (Berlin, Germany) and used without further purification. The nucleotide sequence of NOX-A12 (5′-GCGUGGUGUGAUCUAGAUGUAUUGGCUGAUCCUAGUCAGGUACGC-3′) was obtained from *in vitro* selection experiments as described before^51^. For all cell experiments, Iscove’s Modified Dulbecco’s Media from Life Technologies (Darmstadt, Germanz) was used.

### Preparation of membrane-based surrogate substrates

Glass slides were cleaned following a modified RCA protocol^52^. In brief: the substrates were sonicated in acetone, ethanol, methanol and water for 3 min, subsequently. The samples were immersed in 1:1:5 (v/v/v) H_2_O_2_(30%)/NH_4_OH(25%)/H_2_O and sonicated at room temperature for 3 min, soaked for another 30 min at 60 °C. Substrates were then rinsed 10× with ultrapure water, dried at 70 °C and stored in a vacuum chamber at room temperature. Cell incubation chambers were prepared by bonding bottomless µ-Slide VI^0.4^ channels from Ibidi (Martinsried, Germany) onto microscopic cover slips 256 × 75 mm^2^ (Gerhard Menzel GmbH, Braunschweig, Germany) using polydimethylsiloxane (SYLGARD184, Dow Corning Co., USA).

Lipid stock solutions (in CHCl_3_, 5 mg/mL) were mixed to control the molar fraction of DOGS-NTA (Ni^2+^) or biotin-DOPE in the SOPC matrix. After the solvent evaporation, the lipids were suspended in HBS and sonicated with a S3000 tip sonicator (Misonix Inc., Farmingdale, USA) for 30 min, yielding small unilamellar vesicles (SUVs). The residual titanium particles were removed by centrifugation (Eppendorf, Hamburg, Germany) for 10 min at 13400 g. SUV suspensions were injected into the chamber, incubated for 60 min at 40 °C, and the excess SUVs in supernatant were removed by rinsing with HBS buffer (150 mM NaCl, 10 mM Hepes, pH 7.5). The average lateral distance between lipid anchors <*d*> and thus proteins can be estimated from the molar fraction *c* of lipid anchors by inserting the value of the lipid area of ~65 Å^2 ^^53^:

$$ < \,d\, > \,=\sqrt{\frac{{A}_{lipid}}{c}.}$$

### Coupling of N-cadherin and SDF1α to supported membranes

Prior to the N-cadherin coupling, nitrilotriacetic acid (NTA) headgroups were saturated with Ni^2+^ by incubating spported membranes with 1 mM NiCl_2_ dissolved in HBS (pH 7.5) for 45 min. After replacing the Ni^2+^-containing buffer by the same HBS buffer containing buffer 1 mM CaCl_2_ (pH 7.5), the supported membrane was incubated in 10 µg/mL human recombinant His6 N-cadherin solution for 12 h at room temperature. Prior to the membrane functionalization with SDF1α, supported membranes doped with biotin-DOPE were incubated with 40 µg/mL neutravidin solution for 2 h at room temperature. After rinsing unbound neutravidin with HBS, 10 µg/mL biotinylated SDF1α solution was added. Unbound proteins were removed by rinsing with the medium, and the samples were equilibrated at 37 °C before seeding HSPC.

### Isolation of human HSPC

All samples of primary cells were collected from voluntary donors after obtaining informed consent according to the guidelines approved by the Ethics Committee on the Use of Human Subjects, Heidelberg University. Human HSPC, defined in this study as CD34^+^ cells, were obtained either from umbilical cord blood or from healthy allogeneic stem cell donors. The latter had received a mobilization regimen with G-CSF (10 µg/kg bw per day) for 5 days. The peripheral blood (60 mL) was taken for this study prior to leukapheresis. HSPC were isolated following the previous accounts^10,54^. In brief: mononuclear cells (MNCs) were isolated by density-gradient centrifugation (Merck KGaA, Darmstadt, Germany). CD34^+^ cells from the MNC fraction were enriched by using magnetic beads, followed by 2× sorting steps using an AutoMACS affinity column (all Miltenyi Biotec GmbH, Bergisch-Gladbach, Germany). The cells were allowed to rest for more than 2 h at 37 °C and 5% CO_2_ before use. Follwoing the protocol described by Dexter *et al*.^55^, the cells were stored in long-term bone marrow culture (LTBMC) medium, which consists of 75% Iscove’s modified Dulbecco’s medium (IMDM; Life Technologies Inc., Carlsbad, USA) supplemented with 12.5% FCS, 12.5% horse serum (both Stemcell Technologies Inc., Vancouver, Canada), 2 mM L-glutamine, 100 U/ml penicillin/streptomycin (Life Technologies) and 0.05% hydrocortisone 100 (Sigma–Aldrich Co., St. Louis, USA). Non-viable cells were removed by staining with propidium iodide before the final analysis by flow cytometry, implying the purity of CD34^+^ cells is >95%. Each data point presented was collected from 30–50 cells from 3 donors, and the representable trajectories were shown in each polar plots.

### Cell adhesion experiments

HSPC cell sample in LTBMC were separated into different portions and pre-incubated for 2 h at 37 °C and 5% CO_2_. After the exchanging the medium to the pre-warmed IMDM, HSPC were seeded at a density of 1 × 10^5^ cells/cm^2^ and incubated for 1 h. Prior to recording of adhesion and migration experiments, 5 ng/mL solution of SDF1α or 50 ng/ml or 500 ng/ml solution of synthetic agents (plerixafor or NOXA12) was added to the chamber.

### Live-Cell Imaging

We performed live cell imaging using reflection interference contrast microscopy (RICM) on an Axiobserver (Carl Zeiss AG, Oberkochen, Germany) equipped with a PlanNeofluar Antiflex objective (63×/1.25) with a built-in lambda-quarter plate, a filter cube with crossed polarizers. 20 consecutive images were acquired for at more than 10 positions per condition using an Orca ER CCD camera (Hamamatsu Photonics, Hamamatsu, Japan) with an exposure time of 0.1 s. From the average height and standard deviation of heights in each pixel, adhesion was detected when (a) the pixel lay within the cell contact area enclosed by the interference fringe pattern and (b) the standard deviation of heights was smaller than the noise.

Time-lapse imaging of HSPC migration was performed on a Keyence BZ-9000 (Keyence, Osaka, Japan) equipped with a humidified and temperature controlled chamber. For each experimental condition, we selected 1–2 positions and recorded phase contrast images using a Plan Fluor air objective (40×/0.6) over 6 h (frame rate: 25 mHz). All data sets were analyzed using self-written routines in Matlab 7.7.0 (R2008b) and ImageJ.

## Electronic supplementary material

Supplementary Information
