# Author Correction: Dynamic cellular phenotyping defines specific mobilization mechanisms of human hematopoietic stem and progenitor cells induced by SDF1α versus synthetic agents

**DOI:** 10.1038/s41598-018-25253-7

**Published:** 2018-04-30

**Authors:** Cornelia Monzel, Alexandra S. Becker, Rainer Saffrich, Patrick Wuchter, Volker Eckstein, Anthony D. Ho, Motomu Tanaka

**Affiliations:** 1 Physical Chemistry of Biosystems, Institute of Physical Chemistry, Heidelberg University, 69120 Heidelberg, Germany; 2 Department of Medicine V, Heidelberg University, 69120 Heidelberg, Germany; 3 Institute for IntegratedCell-Material Sciences, Kyoto University, 606-8501 Kyoto, Japan; 4 Present Address: Laboratoire Physico-Chimie, Institut Curie, CNRS UMR168, 75005 Paris, France; 5 Present Address: Institute of Transfusion Medicine and Immunology, Medical Faculty Mannheim, Heidelberg University, German Red Cross Blood Service Baden-Württemberg – Hessen, 68167 Mannheim, Germany

Correction to: *Scientific Reports* 10.1038/s41598-018-19557-x, published online 30 January 2018

The original version of this Article contained a typographical error in the title.

“Dynamic cellular phynotyping defines specific mobilization mechanisms of human hematopoietic stem and progenitor cells induced by SDF1α versus synthetic agents”

now reads:

“Dynamic cellular phenotyping defines specific mobilization mechanisms of human hematopoietic stem and progenitor cells induced by SDF1α versus synthetic agents”

This error has now been corrected in the PDF and HTML versions of the Article.

