# Supplementary material for: Dynamic cellular phenotyping defines specific mobilization mechanisms of human hematopoietic stem and progenitor cells induced by SDF1α versus synthetic agents
Source: Sci Rep. 2018 Jan 30;8:1841. doi: 10.1038/s41598-018-19557-x (PMC5789976; doi:10.1038/s41598-018-19557-x)
Supplement: Supplementary file 1 — Supplementary Information [file 41598_2018_19557_MOESM1_ESM.pdf]

Supporting Information:

Dynamic cellular phenotyping defines specific mobilization mechanisms of human hematopoietic stem and progenitor cells induced by SDF1α versus synthetic agents

Cornelia Monzel[1,§], Alexandra S. Becker[1], Rainer Saffrich[2,#], Patrick Wuchter[2,#], Volker Eckstein[2], Anthony D. Ho[2,*], Motomu Tanaka[1,3,*]

[1] Physical Chemistry of Biosystems, Institute of Physical Chemistry, Heidelberg University, 69120 Heidelberg, Germany

[2] Department of Medicine V, Heidelberg University, 69120 Heidelberg, Germany

[3] Institute for Integrated Cell-Material Sciences, Kyoto University, 606-8501, Kyoto, Japan

[§] present address: Laboratoire Physico-Chimie, Institut Curie, CNRS UMR168, 75005 Paris, France

[#] present address: Institute of Transfusion Medicine and Immunology, Medical Faculty Mannheim, Heidelberg University, German Red Cross Blood Service Baden-Württemberg – Hessen, 68167 Mannheim, Germany

* corresponding authors: anthony_dick.ho@urz.uni-heidelberg.de, tanaka@uni-heidelberg.de

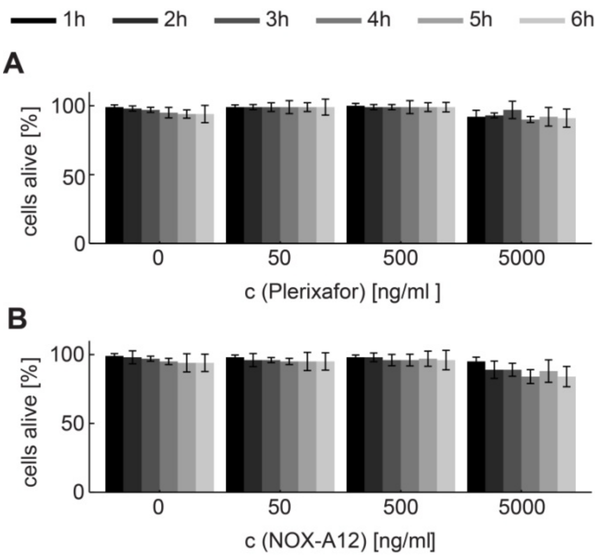

SI Figure 1: Cell viability observed over 6 h for HSPCs adhering to SDF1α substrates with (A) plerixafor and (B) NOX A12 added in solution at different concentrations. The cell viability was assessed by the exclusion test using the diazo dye (trypan blue), following our previous account (Burk et al., Sci. Rep. (2015)).

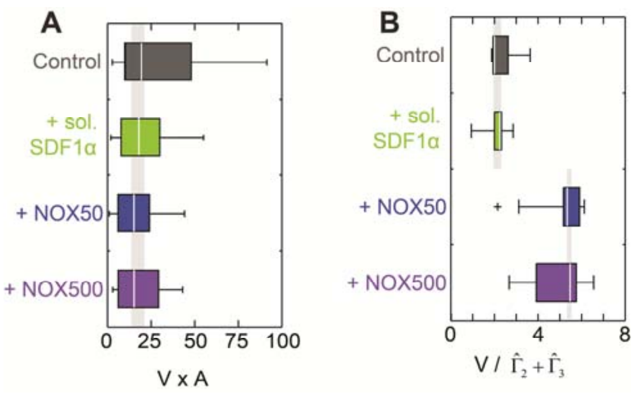

SI Figure 2: Influence of NOX-A12 concentration on (A) $V \times A$ and (B) $V/\Gamma_2 + \Gamma_3$.

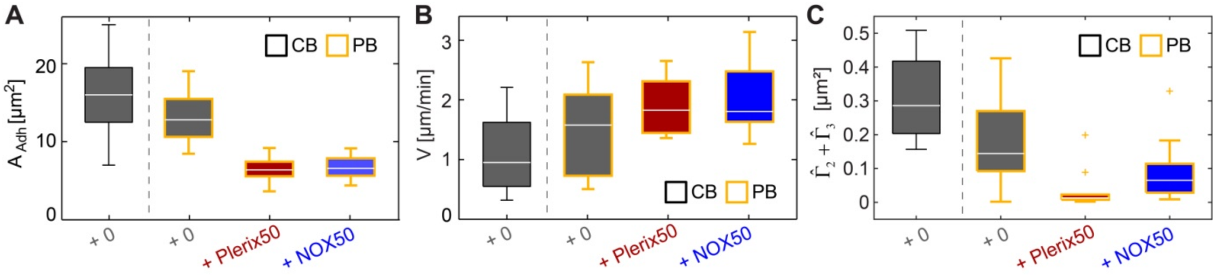

SI Figure 3: Comparison of peripheral blood to cord blood HSPC. (A) cell adhesion area, A_{Adh}, (B) migration velocity, V, and (C) $\hat{\Gamma}_2+\hat{\Gamma}_3$ in absence and presence of plerixafor (Plerix50) or NOX-A12 (NOX50).
